# Comparison of the Cumulative Incidence Rates of Coal Workers’ Pneumoconiosis between 1970 and 2013 among Four State-Owned Colliery Groups in China

**DOI:** 10.3390/ijerph120707444

**Published:** 2015-06-30

**Authors:** Kai Cui, Fuhai Shen, Bing Han, Juxiang Yuan, Xia Suo, Tianbang Qin, Hongbo Liu, Jie Chen

**Affiliations:** 1 School of Public Health, China Medical University, Shenyang 110013, Liaoning, China; E-Mails: cuikai19851022@tom.com (K.C.); shfh600@163.com (F.S.); alex0229@163.com (B.H.); liuhbabc@163.com (H.L.); 2 School of Public Health, Hebei United University, Tangshan 063000, Hebei, China; E-Mail: yuanjx@heuu.edu.cn; 3 Occupational Disease Prevention and Treatment Hospital of Datong Coal Mine Group, Datong 037003, Shanxi, China; E-Mail: suoxsuox@sina.com; 4 Occupational Disease Prevention and Treatment Hospital of Kailuan Colliery Group, Tangshan 063000, Hebei, China; E-Mail: qintbqin@sina.com

**Keywords:** coal workers’ pneumoconiosis, cumulative incidence rate, life-table method

## Abstract

The purpose of this study was to identify differences in the incidence characteristics of coal workers’ pneumoconiosis (CWP) based on data from four large state-owned colliery groups of China, by comparing the cumulative incidence rates of CWP. We investigated 87,904 coal workers from the Datong, Kailuan, Fuxin, and Tiefa Colliery Groups, who were exposed to dust for at least 1 year. The cumulative incidence rate of CWP was calculated with the life-table method and stratified analysis among coal workers with different occupational categories during different years of first dust exposure. Our results showed the cumulative incidence rate of Datong was higher than that of any other colliery group among workers with different occupational categories during different years of first dust exposure. For Datong workers who started their dust exposure in the 1970s, the cumulative incidence rates of CWP among tunneling, mining, combining, and helping workers were 34.77%, 10.20%, 34.59%, and 4.91% during the observed time of 34 years, respectively. For those in the 1980s, the cumulative incidence rates were 32.29%, 13.51%, 2.98%, and 0.47%, respectively. The cumulative incidence rates of Fuxin and Tiefa were the lowest. In conclusion, the Datong colliery has the highest cumulative incidence rate of CWP among the four studied collieries, followed by Kailuan. The cumulative incidence rates of Fuxin and Tiefa were the lowest. Additional dust-proofing measures for decreasing dust concentrations are still necessary.

## 1. Introduction

Coal workers’ pneumoconiosis (CWP) is a slowly progressive lung disease caused by inhalation and deposition of coal mine dust in the lungs, and it is one of the most common occupational diseases worldwide [1,2,3,4]. Although many measures have been used to prevent CWP, it is still an international public health issue [5,6,7]. In the United States, the exposure limit for respirable coal mine dust was established with the enactment of the 1969 Federal Coal Miner Health and Safety Act [7,8]. Since then, the overall prevalence of CWP among coal workers in the United States has declined from 11.2% from 1970 to 1974 to 2.0% during 1995 to 1999; however, since 2000 the prevalence of CWP has increased to 3.33% for 2005 to 2006 [9,10]. In China, the Pneumoconiosis Disease Prevention and Control Regulations of People’s Republic of China were promulgated in the 1980s, and the Law of the People’s Republic of China on Prevention and Control of Occupational Diseases was implemented in 2002 to provide safeguards for the prevention of CWP. Additionally, other preventive methods have been taken, including wet operation in coal mines, improved ventilation, and regular physical examinations of coal workers. However, CWP is still the most widespread occupational disease in China [11,12]. During the period of 1997 to 2009, the number of new pneumoconiosis patients was 122,333, and 44.2% of these patients were diagnosed as CWP [13]. According to the 2013 National Occupational Diseases Report, there were 13955 CWP patients in 2013, accounting for 52.87% of all cases of occupational disease [14].

CWP is an incurable disease, and it not only affects patients and their families but also society as a whole [15]. Today, there are more than six million coal workers employed in China [16], making prevention of CWP an important consideration. Identification of the incidence characteristics of CWP is a key step in its prevention. However, epidemiological studies on CWP in China are limited to certain individual regions or mines, and these studies have reported differing prevalence rates of CWP [17]. With that, it was necessary to compare the rate of CWP among different regions in order to identify the incidence characteristics of the disease. The Datong, Kailuan, Fuxin, and Tiefa colliery groups are all state-owned and located in Northern China. All the colliery groups have experienced similar progress in mining techniques, such as blasting, as well as caving processes. Mechanical mining has been gradually adopted in each colliery group, along with dust-proofing measures, wet operation, and mechanical ventilation. The four colliery groups are representative of the coal mining industry in China as far as their experience in mining processes and dust-proofing efforts is concerned. In the present study, we investigated coal workers who were exposed to coal dust from these four large state-owned colliery groups in China and analyzed the cumulative incidence rate of CWP. We compared the cumulative incidence rates of CWP among workers of the four colliery groups who were first exposed to dust between 1970 and 2013 in order to characterize the incidence of CWP.

## 2. Materials and Methods

### 2.1. Study Population

The study cohort included 2873 CWP patients and 85,031 coal workers without CWP who were registered in the employment records of the Datong, Kailuan, Fuxin, and Tiefa colliery groups. The employment records included personnel files, individual medical records and occupational history records from 1 January 1970 to 31 December 2013. All the included coal workers needed to have been exposed to coal dust for at least 1 year, with exposure starting in 1970. We collected data on work history and diagnosis of pneumoconiosis through 31 December 2013.

The database was constructed with the included demographic details, work history records with the date of dust exposure, individual medical records and pneumoconiosis diagnosis records. The coal workers had physical examinations every 2 years at occupational disease prevention and treatment hospitals of the four colliery groups. The workers must have been offered a chest X-ray approximately every 2 years. All medical records were preserved in each occupational disease prevention and treatment hospital. We collected medical records between 1 January 1970 and 31 December 2013 for workers who started dust exposure in 1970. Information on demographic details and work history were obtained from the human resources department of the four colliery groups. Pneumoconiosis diagnosis records of CWP patients were obtained from the occupational disease prevention and treatment hospital of each colliery group. For coal workers without CWP, the observed years began on the first day of dust exposure and ended on the date of last follow-up or on the study end date (31 December 2013). For CWP patients, the observation period began on the first day of dust exposure and ended on the date when they were diagnosed with CWP.

### 2.2. Diagnosis of Pneumoconiosis

The diagnosis of CWP was based on the Chinese Diagnostic Standard for Pneumoconiosis and corresponding standard films of pneumoconiosis [18]. The Chinese Diagnostic Standard for Pneumoconiosis was developed based on the Guidelines for the Use of the ILO International Classification of Radiographs of Pneumoconiosis from the International Labour Organization (ILO). Both of these diagnostic criteria are based on the same diagnosis judgment principle. Five qualified experts who were all members of the Pneumoconiosis Diagnosis Committee independently read the chest radiographs of CWP patients and other investigated workers. If there was difference among the 5 experts on the diagnosis, the diagnosis judgment principle was that the minority should be subordinate to the majority. Using the ILO criteria, pneumoconiosis was classified as stage I, II, or III in the Chinese diagnostic criteria. The definition of stage I pneumoconiosis is that the total profusion of small opacities is grade 1 and the distribution reaches at least two lung zones. The definition of stage II pneumoconiosis is that the total profusion of small opacities is grade 2 and the distribution reaches more than four lung zones, or that the total profusion of small opacities is grade 3 and distribution reaches four lung zones. Stage III pneumoconiosis is defined as the total profusion of small opacities is grade 3 and the distribution reaches more than four lung zones, or the large opacities are at least 20 μm long and 10 μm wide [19].

### 2.3. Occupational Categories

We defined four working areas in the underground mines by reviewing the working history of the workers: tunneling, mining, combining, and helping [20,21,22]. Workers who consistently worked in the same area were defined by their respectively work area titles: tunneling, mining, combining, or helping. The duration of dust exposure for each coal worker was the sum of years of each dust exposure job. The duration of each dust exposure job was measured from the start date to the end date of the job. Coal workers who had a duration of work exposure in a tunneling area for more than half of the whole duration of dust exposure were classified as tunneling; this included pneumatic drilling, blasting, and lashing of hard rock materials to create tunnels. If workers’ duration in a tunneling area was less than 2 years and that in mining areas was more than half of the whole duration, they were classified as mining (drilling, blasting, cutting, and loading of the coal). Both tunneling and mining miners were in direct contact with dust producing areas. Combining workers were those whose duration in tunneling areas was more than 2 years but not more than half of the entire duration of dust exposure. The workers who could not be included in tunneling, mining, or combining categories were classified as helping, (maintenance, transportation, washing plant and cinders workers). The helping workers did not have direct contact with the dust producing areas of mining or tunneling.

### 2.4. Statistical Analysis

Four subcohorts were established according to occupational category: tunneling, mining, combining and helping. The cumulative incidence rate of CWP in the corresponding observed years was calculated using the life table method and stratified analysis. We analyzed the cumulative incidence rate of CWP for the workers with different years of first dust exposure (1970s and 1980s) during the 34-year observation period; and for coal workers with different occupational categories during the 44-year observation period. The 34-year cumulative incidence rates in the different occupational categories during different years of first dust exposure were also analyzed after adjusting for the duration of dust exposure (classified by <20 years and ≥20 years). Peto’s log-rank test and *P*-values with Bonferroni correction were used to compare differences in the cumulative incidence rates among the 4 colliery groups [20,21,22]. SPSS 20.0 (SPSS Institute, Inc., Chicago, IL, USA) was used for all statistical procedures. *P* < 0.05 was considered to be statistically significant.

## 3. Results

### 3.1. Demographic Details

The number of workers with and without CWP in each colliery group is shown in Table 1.

**Table 1:** Characteristics of coal workers at the 4 colliery groups.

Characteristics		Datong		Kailuan		Fuxin		Tiefa	
		With CWP	Without CWP	With CWP	Without CWP	With CWP	Without CWP	With CWP	Without CWP
**N**		1847	43,742	838	16,185	143	10,230	45	14,874
**Average durations of dust exposure**		19.8 ± 7.2	18.9 ± 9.5	24.9 ± 7.1	23.0 ± 10.4	23.5 ± 5.1	17.8 ± 10.7	20.5 ± 6.2	17.5 ± 7.7
**Occupational categories**	**Tunneling**	735 (21.82) ^*^	2634	248 (17.91)	1137	96 (2.58)	3630	36 (0.67)	5328
	**Mining**	619 (5.74)	10,161	245 (8.74)	2559	41 (1.54)	2613	5 (0.17)	2921
	**Combining**	297 (5.92)	4721	259 (11.35)	2022	5 (0.60)	822	2 (0.30)	654
	**Helping**	196 (0.74)	26,226	86 (0.81)	10,467	1 (0.03)	3165	2 (0.03)	5971
**Years of first dust exposure**	**1970s**	1217 (17.21)	5854	710 (10.81)	5855	103 (5.56)	1750	36 (4.31)	800
	**1980s**	630 (1.64)	37,888	128 (1.22)	10,330	40 (0.47)	8480	9 (0.06)	14,074

^*^: The numbers with parentheses were the incidence rates of CWP among workers with different occupational categories or different years of first dust exposure.

The incidence rates of CWP were 4.06%, 4.92%, 1.38%, and 0.30% for the Datong, Kailuan, Fuxin, and Tiefa collieries, respectively. Among workers with CWP, the average duration of dust exposure from Datong was lower compared with the other colliery groups. The incidence rates of CWP among workers with different occupational categories were also calculated. Among tunneling workers, the CWP incidence rate at Datong was higher than at other collieries. However, among other occupational categories, Kailuan had the highest rates of CWP. Among workers who started dust exposure in either the 1970s or 1980s, the CWP incidence rate at Datong was higher than at other collieries.

### 3.2. Cumulative Incidence Rates of CWP

**Figure 1:**
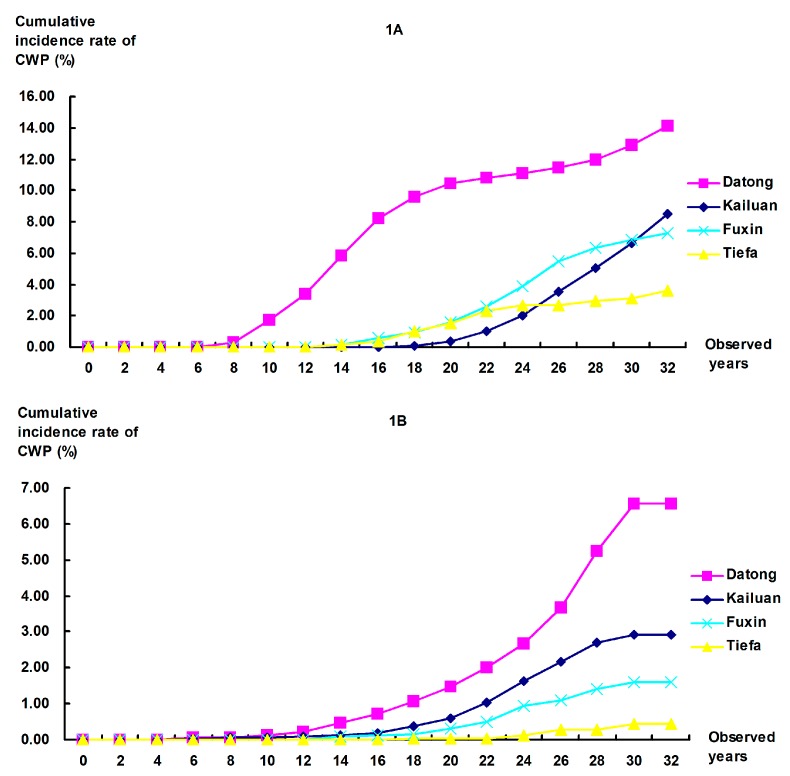
Comparison of cumulative incidence rates of CWP among coal workers with different years of first dust exposure at the four colliery groups. (**A**) Workers who started dust exposure in the 1970s: Significant difference were found in comparisons between any pair of collieries at *P* = 0.0083 (with Bonferroni correction), except for Kailuan *vs.* Fuxin (*P* = 0.016); (**B**) Workers who started dust exposure in the 1980s: All *P*-values for comparisons between colliery groups were <0.001.

During the same 34-year observation period, the cumulative incidence rates of CWP among coal workers with different years of first dust exposure at the four colliery groups were calculated (Figure 1). For the coal workers with first dust exposure in the 1970s, the 34-year cumulative incidence rates of CWP were 14.11%, 8.49%, 7.26%, and 3.62% for Datong, Kailuan, Fuxin, and Tiefa, respectively.

The cumulative incidence rate at Datong was significantly higher than the other collieries, and a similar situation was found among workers who started dust exposure in the 1980s. In the 1980s cohort, the 34-year cumulative incidence rates were 6.56%, 2.91%, 1.62%, and 0.43% for the four colliery groups, respectively. These results indicate that Datong had a higher level of CWP than the other three collieries.

**Figure 2:**
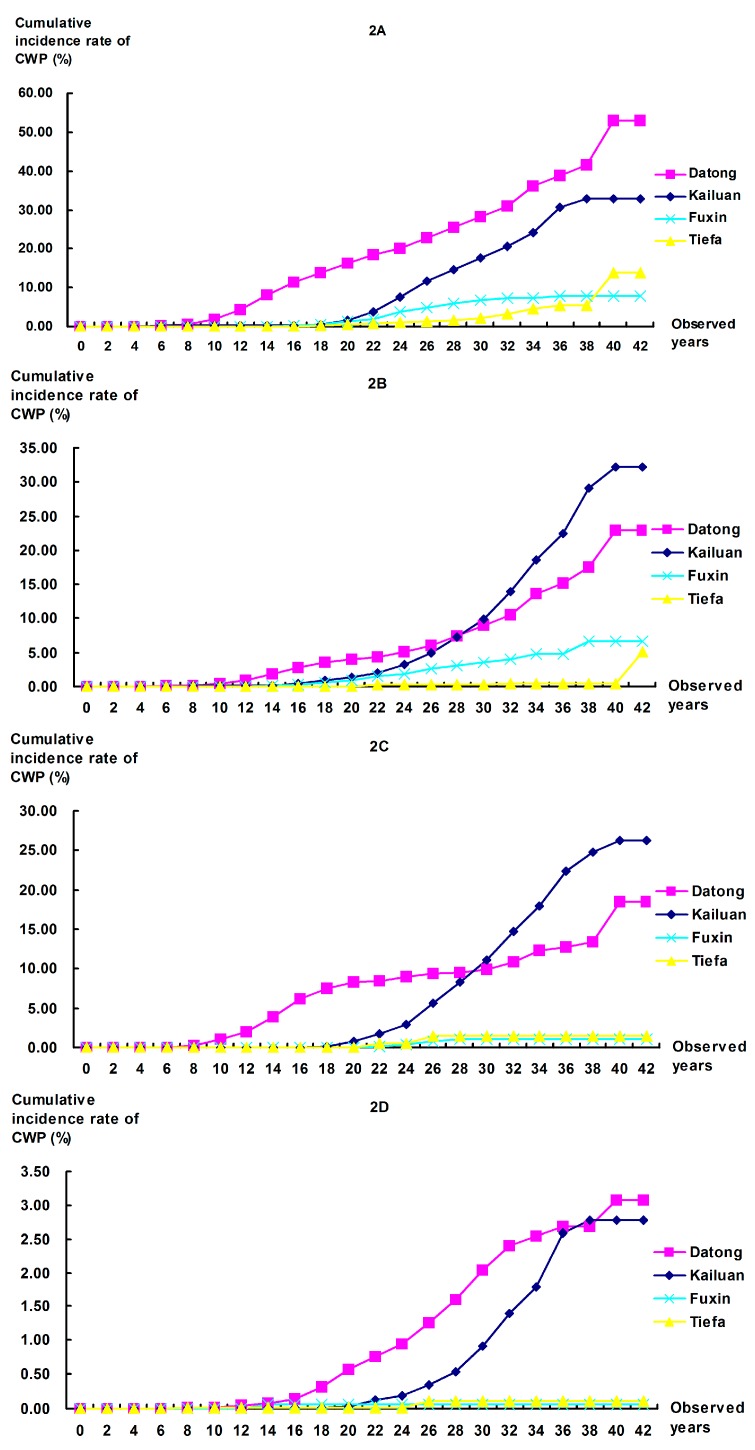
Comparison of cumulative incidence rates of CWP among workers with different occupational categories at the four colliery groups. (**A**) Tunneling workers: All *P*-values for comparisons between colliery groups were <0.001; (**B**) Mining workers: Significant differences were found in any pair of colliery groups (*P* < 0.001), except for Datong *vs.* Kailuan (*P* = 0.134); (**C**) Combining workers: All *P*-values for comparisons between collieries were <0.001, except for Datong *vs.* Kailuan (*P* = 0.021) and Fuxin *vs.* Tiefa (*P* = 0.682); (**D**) Helping workers: Significant differences were found for any comparison between collieries at *P* = 0.0083, except for Kailuan *vs.* Tiefa (*P* = 0.013) and Fuxin *vs.* Tiefa (*P* = 0.969).

**Figure 3:**
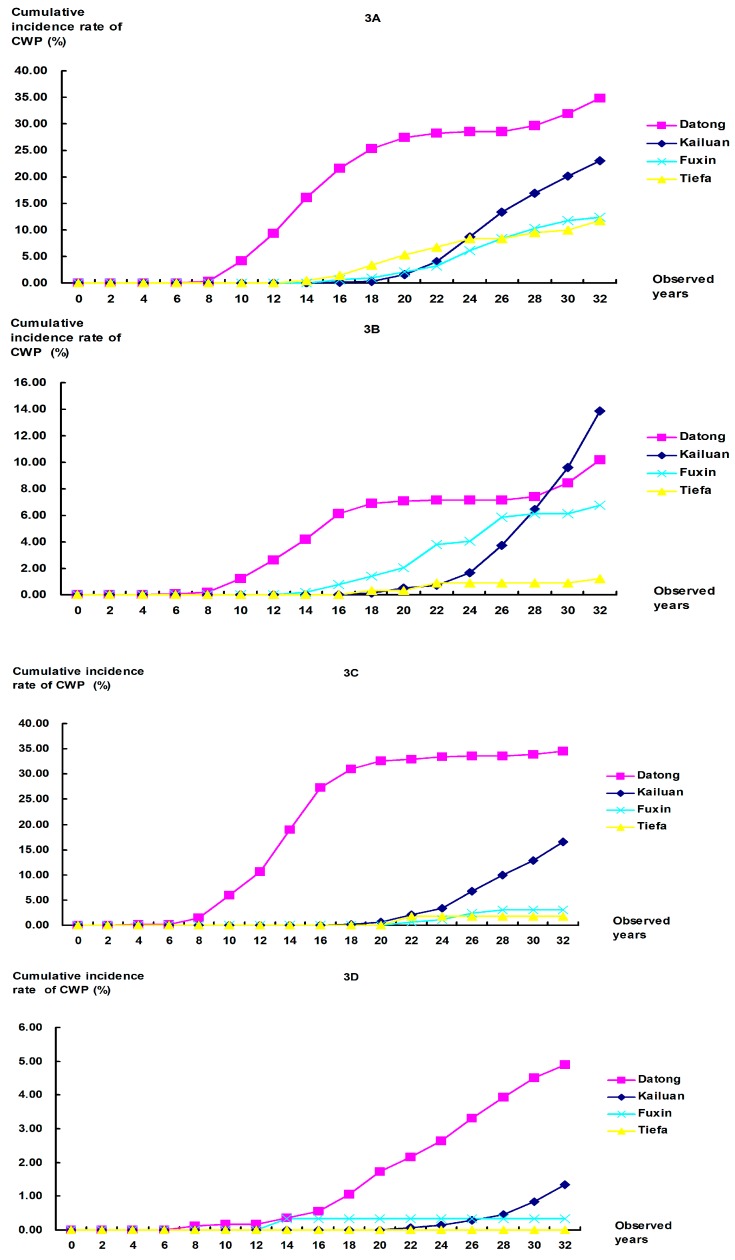
Comparison of cumulative incidence rates of CWP among workers with different occupational categories in the 1970s at the four colliery groups. (**A**) Tunneling workers: Significant differences were found for any comparisons between collieries at *P* = 0.0083, except for Fuxin *vs.* Tiefa (*P* = 0.298); (**B**) Mining workers: All *P*-values for comparisons between collieries were <0.001; (**C**) Combining workers: All *P*-values for comparisons between collieries were <0.001, except for Fuxin *vs.* Tiefa (*P* = 0.565); (**D**) Helping workers: Significant differences were found between Datong and any other colliery (*P* < 0.001), but not for Kailuan *vs.* Fuxin (*P* = 0.074), Kailuan *vs.* Tiefa (*P* = 0.054), or Fuxin *vs.* Tiefa (*P* = 0.532).

**Figure 4:**
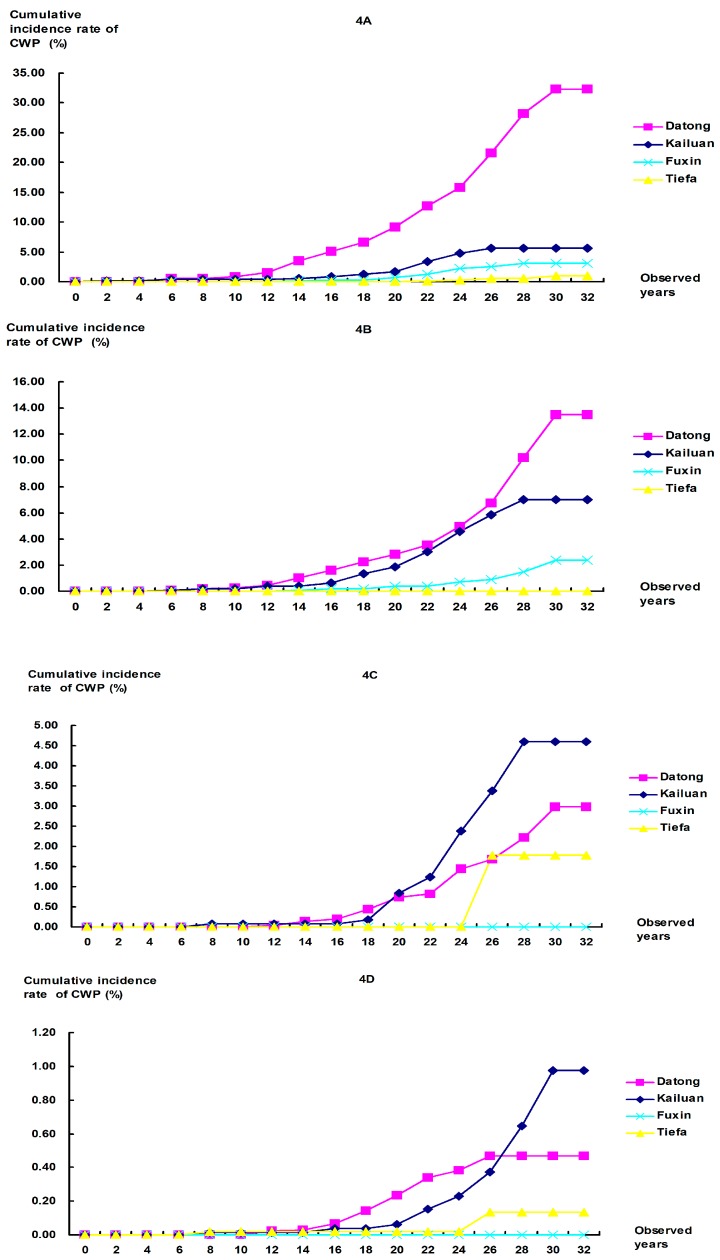
Comparison of cumulative incidence rates of CWP among workers with different occupational categories in the 1980s at the four colliery groups. (**A**) Tunneling workers: Significant differences were found between any pair of collieries at *P* = 0.0083; (**B**) Mining workers: Significant differences were found between any pair of collieries, except for Datong *vs.* Kailuan (*P* = 0.046); (**C**) Combining workers: All *P*-values for comparisons between collieries were <0.001, except for Datong *vs.* Kailuan (*P* = 0.063), Datong *vs.* Tiefa (*P* = 0.016), Kailuan *vs*. Tiefa (*P* = 0.089), and Fuxin *vs.* Tiefa (*P* = 0.366); (**D**) Helping workers: Significant differences were found between any pair of collieries, except for Datong *vs.* Kailuan (*P* = 0.162), Kailuan *vs.* Fuxin (*P* = 0.026), Kailuan *vs.* Tiefa (*P* = 0.062), and Fuxin *vs.* Tiefa (*P* = 0.273).

The 44-year cumulative incidence rates for CWP for tunneling, mining, combining, and helping workers are shown in Figure 2. For tunneling workers, the cumulative incidence rates were 53.05%, 32.84%, 7.90%, and 13.81% for the Datong, Kailuan, Fuxin and Tiefa collieries. For mining workers, the 44-year cumulative incidence rates were 22.97%, 32.22%, 6.68%, and 5.05%, respectively. For combining workers, the cumulative incidence rate at Kailuan was the highest and significantly different from the other collieries, except for Datong; these rates were 18.43%, 26.21%, 1.09%, and 1.43% for Datong, Kailuan, Fuxin, and Tiefa, respectively. Although the cumulative incidence rates at Datong were lower than Kailuan in the cohorts of mining and combining workers, there were no significant differences between the two collieries. For helping workers, the cumulative incidence rates were 3.08%, 2.78%, 0.11%, and 0.06%, respectively. Generally, these results still showed the level of CWP in Datong to be higher compared with the other collieries.

To further analyze the cumulative incidence rate of CWP, workers were classified into several subcohorts with different occupational categories and different years of first dust exposure. The cumulative incidence rates of CWP were calculated among each subcohorts, after adjusting for the duration of dust exposure (Figure 3 and Figure 4). In most subcohorts, CWP cumulative incidence rates at Datong were significantly higher than at other colliery groups during the same observed time of 34 years. In mining workers of the 1970s and combining and helping workers of the 1980s, the rates at Kailuan were higher than those at Datong, but statistical difference was only found in the former subcohort. The rates at Fuxin and Tiefa were lower than those at Datong and Kailuan, and there were no differences between Fuxin and Tiefa in most subcohorts. Similar with that mentioned above, the results found the rate of CWP at Datong to be higher than Kailuan, Fuxin, or Tiefa. The CWP rates at Fuxin and Tiefa were the lowest among the four collieries.

## 4. Discussion

In our present study, we compared the cumulative incidence rate of CWP among four state-owned colliery groups in China. After controlling for the potential influence of occupational categories and years of first dust exposure, the cumulative incidence rate of CWP at Datong colliery were the highest among the four colliery groups, followed by Kailuan. The cumulative incidence rates of Fuxin and Tiefa were the lowest. Additionally, we noted that CWP patients at Datong had shorter durations of dust exposure, suggesting that CWP was a more serious problem at Datong than at other collieries.

The Datong colliery has a mining history of more than 100 years and is also one of the largest coal mines in China. In 2013, the raw coal production at Datong was 132.67 million tons, while production at Kailuan, Fuxin, and Tiefa was 83.54, 27.33 and 25.97 million tons, respectively [23]. Even in 2013, the preventive measures of Datong were not sufficiently effective. Although dust-proofing methods, such as wet operation and mechanical ventilation, were used in Datong, it still had the highest dust concentration among the four collieries. According to monitoring data from Department of Dust Detection and Monitoring at the Datong Colliery Group, the average dust concentration in tunneling areas of Datong was 156.4 mg/m^3^ in the 1980s. During the same period, the average dust concentration in tunneling areas of Fuxin was 47.69 mg/m^3^ [24].

Compared with Datong, Tiefa colliery was established later, which was formally founded in the late 1950s. Since then, wet operation and ventilation devices were used to decrease dust concentration. Further improvements for the reduction of dust concentration were adopted during the 1970s and 1980s and were effective in decreasing worker exposure to dust. Although the Fuxin colliery also has a history of mining for over 100 years, the majority of Fuxin’s mines had been gradually depleted since the 1980s. These conditions influenced the incidence of CWP, and our results showed that the cumulative incidence rates of Tiefa and Fuxin were lower than that of Datong. Additionally, the coal of Datong had higher percentage of carbon and lower volatility than the other mines; namely, the coal rank of Datong was higher than that of Kailuan, Fuxin, and Tiefa. A strong correlation between exposure to higher ranked coal and pneumoconiosis was identified by several studies from different countries [25,26], and it has also resulted in a high cumulative incidence rate of CWP in Datong.

The present study has several limitations. First, data on the working history and diagnosis of pneumoconiosis were collected from the human resources department and the occupational disease preventive and treatment hospital of each colliery. The detailed dust concentration was not collected in this study, something that should be addressed in future studies. However, we did collected data on the working history of each worker with or without CWP, such as occupational categories, years of first dust exposure, and duration of dust exposure, which could indirectly reflect the level of dust exposure for workers. Second, we did not analyze the status of other diseases or causes of death among workers or CWP patients, because we conducted this retrospective study to compare the rate of CWP among the 4 colliery groups. Again, this should be addressed in future studies. Third, to ensure the accuracy and authenticity of data, we only chose workers from four state-owned mines and not those at non-state owned mines. The four large state-owned colliery groups included in our study are representative of the coal mining industry in northern of China. Those private or non-state owned coal mines are not predominant producers of coal nor producers at the same scale or yields as state-owned mines in China. Our aims are to explore the rate of CWP in the coal industry of China using data from four large state-owned coal enterprises. Our results call for improved dust-proofing measures to decrease the incidence of CWP among workers.

## 5. Conclusions

In this study, we found that Datong had the highest cumulative incidence rate of CWP among the 4 collieries, followed by Kailuan. The cumulative incidence rates of Fuxin and Tiefa were the lowest. To control CWP, it is necessary to further enhance comprehensive dust-proofing measures and decrease the dust concentration in working areas of coal mines in China.
